# Tobacco Use in Top-Grossing Movies — United States, 2010–2016

**DOI:** 10.15585/mmwr.mm6626a1

**Published:** 2017-07-07

**Authors:** Michael A. Tynan, Jonathan R. Polansky, Kori Titus, Renata Atayeva, Stanton A. Glantz

**Affiliations:** ^1^Office on Smoking and Health, National Center for Chronic Disease Prevention and Health Promotion, CDC; ^2^Onbeyond LLC, Fairfax, California; ^3^Breathe California of Sacramento-Emigrant Trails, Sacramento, California; ^4^University of California, San Francisco.

The Surgeon General has concluded that there is a causal relationship between depictions of smoking in the movies and the initiation of smoking among young persons (*1*). The more youths see smoking on screen, the more likely they are to start smoking; youths who are heavily exposed to onscreen smoking imagery are approximately two to three times as likely to begin smoking as are youths who receive less exposure (*1*,*2*). A *Healthy People 2020* objective is to reduce the proportion of youths exposed to onscreen tobacco marketing in movies and television (Tobacco Use Objective 18.3) (*3*). To assess the recent extent of tobacco use imagery in youth-rated movies (G, PG, PG-13*), 2010–2016 data from Thumbs Up! Thumbs Down! (TUTD), a project of Breathe California of Sacramento-Emigrant Trails were analyzed and compared with previous reports.^†^ In 2016, 41% of movies that were among the 10 top-grossing movies in any calendar week included tobacco use, compared with 45% in 2010. Among youth-rated movies, 26% included tobacco use in 2016 (including 35% of PG-13 movies) compared with 31% in 2010 (including 43% of PG-13 movies). The steady decline in the number of tobacco incidents in youth-rated movies from 2005–2010 stopped after 2010. The total number of individual occurrences of tobacco use in a movie (tobacco incidents) in top-grossing movies increased 72%, from 1,824 in 2010 to 3,145 in 2016, with an increase of 43% (from 564 to 809) occurring among PG-13 rated movies. Reducing tobacco use in youth-related movies could help prevent the initiation of tobacco use among young persons.

TUTD counts occurrences of tobacco incidents, defined as the use or implied use of a tobacco product (cigarettes, cigars, pipes, hookah, smokeless tobacco products, and electronic cigarettes) by an actor, in U.S. top-grossing movies each year. Trained monitors count all tobacco incidents in those movies that are among the 10 top-grossing movies in any calendar week of the year. Previous reports have used this criterion because U.S. movies ranked in the 10 top-grossing movies for at least 1 week have accounted for 96% of U.S. ticket sales (*4*–*6*). At least two monitors independently evaluate each film; any differences are resolved by a supervisor who independently watches the film using the same protocol. Incidents of implied use have been rare and occur when a person is handed or is holding, but does not necessarily use, a tobacco product. A new incident was counted each time 1) a tobacco product went off screen and then came back on screen; 2) a different actor was shown with a tobacco product; or 3) a scene changed and the new scene contained the use or implied use of a tobacco product.^§^

To calculate the percentage of movies with tobacco incidents, the number of movies with tobacco incidents was divided by the total number of movies, and the average number of tobacco incidents per movie was calculated for each motion picture company. For each year during 2010–2016, the number of top-grossing movies with tobacco incidents and overall number of tobacco incidents were calculated. Results were also analyzed by Motion Picture Association of America (MPAA) ratings (G, PG, PG-13, R). Findings were also compared with data from reports from 1991–2010 (*4*,*5*).

In 2016, among 143 top-grossing movies, 59 (41%) had tobacco incidents, compared with 62 (45%) of 137 in 2010; among top-grossing R-rated movies, 35 (67%) of 52 had tobacco incidents in 2016, compared with 35 (71%) of 49 in 2010 (Table 1). Among youth-rated movies (G, PG, or PG-13), 24 (26%) of 91 had tobacco incidents in 2016, compared with 27 (31%) of 88 in 2010. Overall, from 2010 to 2016, the number of top-grossing movies with tobacco incidents ranged from 58 in 2014 to 76 in 2013 (Table 1).

**TABLE 1:** Number and percentage of top-grossing movies with any tobacco incidents, by Motion Picture Association of America (MPAA) rating and movie company — United States, 2010–2016

Movie company	MPAA rating*	No. (%)							
		2010	2011	2012	2013	2014	2015	2016	Total
Comcast (Universal)	G/PG	0 (0)	0 (0)	0 (0)	0 (0)	0 (0)	0 (0)	0 (0)	**0 (0)**
	PG-13	1 (17)	4 (40)	3 (50)	2 (29)	6 (67)	3 (30)	2 (18)	**21 (36)**
	R	6 (86)	6 (86)	8 (73)	10 (77)	5 (71)	5 (50)	2 (22)	**42 (66)**
Disney	G/PG	1 (11)	0 (0)	0 (0)	0 (0)	0 (0)	0 (0)	0 (0)	**1 (2)**
	PG-13	0 (0)	3 (60)	1 (33)	2 (40)	0 (0)	2 (50)	1 (20)	**9 (32)**
	R	0 (0)	1 (100)	0 (0)	1 (100)	0 (0)	0 (0)	0 (0)	**2 (100)**
Fox	G/PG	0 (0)	2 (29)	1 (17)	0 (0)	0 (0)	0 (0)	0 (0)	**3 (7)**
	PG-13	3 (38)	3 (50)	2 (40)	2 (33)	4 (57)	4 (36)	4 (67)	**22 (45)**
	R	5 (71)	2 (100)	3 (100)	6 (100)	5 (63)	5 (100)	4 (80)	**30 (83)**
Independents^†^	G/PG	3 (60)	0 (0)	1 (50)	2 (67)	1 (20)	2 (67)	1 (17)	**10 (37)**
	PG-13	6 (55)	6 (46)	12 (52)	10 (50)	9 (47)	10 (59)	6 (38)	**59 (50)**
	R	15 (83)	6 (67)	15 (68)	19 (83)	7 (58)	16 (70)	16 (70)	**94 (72)**
Sony	G/PG	0 (0)	1 (17)	1 (33)	1 (33)	2 (50)	1 (20)	0 (0)	**6 (24)**
	PG-13	8 (67)	7 (58)	6 (60)	4 (57)	5 (71)	3 (50)	3 (33)	**36 (57)**
	R	2 (67)	7 (78)	6 (75)	5 (83)	5 (83)	4 (100)	5 (100)	**34 (83)**
Time Warner (Warner Bros.)	G/PG	0 (0)	0 (0)	0 (0)	1 (100)	0 (0)	0 (0)	0 (0)	**1 (8)**
	PG-13	2 (22)	4 (33)	4 (44)	3 (27)	2 (25)	4 (50)	2 (20)	**21 (31)**
	R	4 (50)	3 (50)	5 (83)	3 (50)	3 (33)	6 (60)	4 (67)	**28 (55)**
Viacom (Paramount)	G/PG	0 (0)	3 (60)	0 (0)	0 (0)	0 (0)	0 (0)	0 (0)	**3 (23)**
	PG-13	3 (75)	3 (50)	2 (40)	1 (25)	2 (25)	2 (67)	5 (56)	**18 (46)**
	R	3 (50)	1 (33)	3 (75)	4 (100)	2 (67)	2 (67)	4 (100)	**19 (70)**
Subtotal by ratings	All G/PG	4 (11)	6 (14)	3 (11)	4 (21)	3 (12)	3 (13)	1 (4)	**24 (13)**
	All PG-13	23 (43)	30 (47)	30 (49)	24 (40)	28 (46)	28 (47)	23 (35)	**186 (44)**
	All youth-rated^§^	27 (31)	36 (37)	33 (37)	28 (35)	31 (36)	31 (38)	24 (26)	**210 (34)**
	All R	35 (71)	26 (70)	40 (74)	48 (81)	27 (60)	38 (69)	35 (67)	**249 (71)**
**All ratings**		**62 (45)**	**62 (46)**	**73 (51)**	**76 (55)**	**58 (44)**	**69 (50)**	**59 (41)**	**459 (51)**

* G = General Audiences (all ages admitted); PG-13 = Parents Strongly Cautioned (some material might be inappropriate for preteenagers); R = Restricted (under age 17 requires accompanying parent or adult guardian).

^†^ Independent movie companies include producer-distributors that are not members of MPAA, but regularly adhere to MPAA ratings and advertising rules.

^§^ Youth-rated includes G/PG and PG-13.

Although the percentage of top-grossing movies with tobacco incidence decreased during 2010–2016, the total number of tobacco incidents in top-grossing movies increased by 72%, from 1,824 to 3,145 (Table 2). The total number of incidents in G or PG movies decreased by 87% (from 30 to 4), whereas the number in PG-13 movies increased 43% (from 564 to 809), and the number in R-rated movies increased 90% (from 1,230 to 2,332). Compared with previous studies (*4*,*5*), smoking incidents had peaked at 3,962 incidents in 2005; the year with the lowest number of recorded smoking incidents (1,613) was 1998 (Figure). During 2010–2016, the lowest number of tobacco incidents (1,743) occurred in 2015; the highest number since 2010 (3,145) occurred in 2016, representing an 80% increase compared with the previous year. 

**TABLE 2:** Number of tobacco incidents in top-grossing movies, by Motion Picture Association of America (MPAA) rating and movie company — United States, 2010–2016

Movie company	MPAA rating*	2010	2011	2012	2013	2014	2015	2016	Total
Comcast (Universal)	G/PG	0	0	0	0	0	0	0	**0**
	PG-13	19	78	39	53	173	11	266	**639**
	R	35	154	251	398	76	113	50	**1,077**
Disney	G/PG	10	0	0	0	0	0	0	**10**
	PG-13	0	148	102	57	0	123	6	**436**
	R	0	20	0	4	0	0	0	**24**
Fox	G/PG	0	3	2	0	0	0	0	**5**
	PG-13	96	174	205	3	101	150	145	**874**
	R	274	36	47	278	210	59	47	**951**
Independents^†^	G/PG	20	0	19	2	15	5	4	**65**
	PG-13	132	22	282	315	625	187	128	**1,691**
	R	582	216	720	511	559	456	889	**3,933**
Sony	G/PG	0	9	2	1	12	83	0	**107**
	PG-13	198	166	178	26	184	15	144	**911**
	R	33	537	246	155	225	156	576	**1,928**
Time Warner (Warner Bros.)	G/PG	0	0	0	5	0	0	0	**5**
	PG-13	4	106	265	309	16	30	40	**770**
	R	80	62	267	233	343	322	541	**1,848**
Viacom (Paramount)	G/PG	0	95	0	0	0	0	0	**95**
	PG-13	115	50	92	12	66	3	80	**418**
	R	226	4	166	217	34	30	229	**906**
Subtotals by ratings	All G/PG	30	107	23	8	27	88	4	**287**
	All PG-13	564	744	1,163	775	1,165	519	809	**5,739**
	All youth-rated^§^	594	851	1,186	783	1,192	607	813	**6,026**
	All R	1,230	1,029	1,697	1,796	1,447	1,136	2,332	**10,667**
**All ratings**		**1,824**	**1,880**	**2,883**	**2,579**	**2,639**	**1,743**	**3,145**	**16,693**

* G = General Audiences (all ages admitted); PG-13 = Parents Strongly Cautioned (some material might be inappropriate for preteenagers); R = Restricted (under age 17 requires accompanying parent or adult guardian).

^†^ Independent movie companies include producer-distributors that are not members of MPAA, but regularly adhere to MPAA ratings and advertising rules.

^§^ Youth-rated includes G/PG and PG-13.

**FIGURE:**
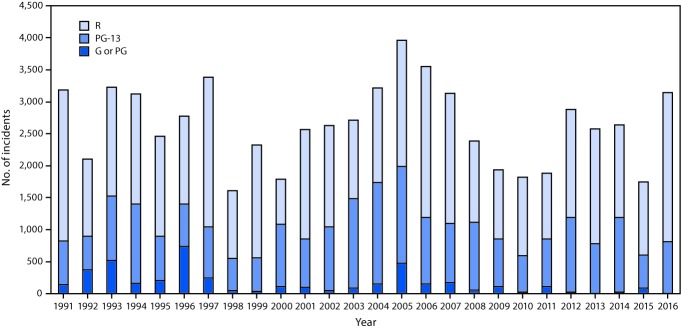
Tobacco incidents in top-grossing movies, by movie rating* — United States, 1991–2016 *Ratings are assigned by the Motion Picture Association of America, the trade organization that represents the six major movies studios. G = General Audiences (all ages admitted); PG = Parental Guidance Suggested (some material might not be suitable for children); PG-13 = Parents Strongly Cautioned (some material might be inappropriate for children under 13); and R = Restricted (under 17 requires accompanying parent or adult guardian).

## Discussion

The findings in this report indicate that although there were previously reported declines in the number of youth-rated movies with tobacco incidents observed during 2005–2010 (*4*,*5*), since 2010 there has been no progress in reducing the total number of tobacco incidents in youth-rated movies. Had the trend established from 2005 to 2010 continued, all youth-rated films would have been smoke-free by 2015. Although there were fewer top-grossing movies depicting tobacco use in 2016 compared with 2010, an increase in the number of such incidents occurred, thereby concentrating exposure to tobacco use in fewer films. The average number of tobacco incidents increased 55% in youth-rated movies with any tobacco depiction, from 22 incidents in 2010 to 34 incidents in 2016, and increased 91% in R-rated films with any tobacco depictions, from 35 incidents in 2010 to 67 incidents in 2016. Tobacco use depictions are now uncommon in G and PG films; however, the 43% increase in the total number of tobacco-use incidents in PG-13 movies, from 564 in 2010 to 809 in 2016, is of particular public health concern because of the established causal relationship between youths’ exposure to smoking in movies and smoking initiation (*1*).

The six major motion picture companies have policies to reduce depictions of tobacco use in youth-rated films,^¶^ which likely contributed to the reduction in the number of movies with tobacco incidents during 2005–2010. TUTD started systematic data collection of onscreen tobacco use in movies in 1991. Occurrences of tobacco use in movies varied from 1991 to 2010, reaching a peak in 2005 then declining by almost half by 2010 (*4*,*5*). Public health organizations, investors, state health departments, and state attorneys general raised concerns regarding tobacco incidents in movies beginning in 2001, which might account, in part, for the decrease in onscreen tobacco incidents after 2005 and before major motion picture companies adopted policies regarding tobacco imagery in youth-rated films (*4*,*5*). However, the lack of progress in recent years suggests that enhanced measures to address tobacco incidents in movies are warranted.

One such intervention would be the assignment of an R rating to any movie with smoking or other tobacco-use imagery (unless the portrayal is of actual historical figures who smoked, a documentary, or if the portrayal includes the negative effects of tobacco use) (*7*–*9*). Other interventions include certifying that no payments have been received by the studio or producers for depicting tobacco use in the movies and ending the onscreen depiction of actual tobacco brands (*7*,*8*). These and additional interventions, if implemented, could help eliminate tobacco incidents in youth-rated movies (*7*–*9*). State and local health departments could also work with state agencies that manage movie subsidies to ensure that such subsidies do not go to films that include depictions of tobacco use. During 2010–2016, approximately 24 states awarded approximately $3.5 billion in public subsidies, such as tax credits, to productions of movies with tobacco incidents, including youth-rated movies.**

Currently the MPAA does not assign R ratings to movies based on tobacco use incidents. In 2007, the MPAA developed a smoking “rating descriptor” that is applied to a few movies that contain smoking. These descriptors can appear in fine print in the box with the letter rating for a movie and can appear on advertisements and promotions to describe the type of content in a movie, such as language, violence, nudity, or sexual content. However, 89% of top-grossing, youth-rated movies with smoking did not carry the MPAA “smoking descriptor” in 2015 (*9*). A longitudinal cohort study of smoking onset among youths viewing movies released during 1998–2003 concluded that classifying movies with smoking with an R rating could reduce the number of teen smokers by approximately 18% (*7*). The Surgeon General notes that the magnitude of the effect of an R rating for smoking would be similar to increasing the price of cigarettes from $6.00 to $7.50 per pack (*10*).

The findings in this report are subject to at least three limitations. First, detailed audience composition data are not publicly available; therefore, the number of tobacco use impressions (one person seeing one tobacco incident one time, a measure of total audience exposure) delivered by a particular movie to children and adolescents could not be determined. Second, the sample did not include all movies. However, the samples of top grossing movies were used because they are expected to account for approximately 95% of theater tobacco-use impressions (*4*–*6*). Finally, the measure used to assess tobacco exposure from movies should be interpreted cautiously because movies can be viewed through other channels (e.g., recorded media, such as DVDs and Blu-ray; television; and online streaming) that do not contribute to the calculation of in-theater impressions. As viewing platforms expand, it is important to identify whether youths are being exposed to tobacco imagery through other media sources, such as broadcast and cable television, on-demand services, and social media. Further research into youths’ exposure to tobacco imagery in these and other forms of media could also help identify the impact that exposure through these sources has on youths’ tobacco use.

If current trends continue, 5.6 million youths who are alive today are projected to die from tobacco-related diseases (*10*). Whereas the number of top-grossing movies with tobacco use incidents continued to decline from 2010 to 2016, one in four youth-rated movies featured tobacco imagery, which is harmful to youths and causes youths to start using tobacco. The frequency and increase in tobacco incidents in PG-13 movies is of public health concern because these movies are rated as appropriate for youths. Opportunities exist for movie studios to reduce tobacco incidents that appear in youth-related movies, including rating films with smoking R, which would help prevent or delay the initiation of tobacco use among young persons and prevent premature deaths from tobacco-related diseases.

**Summary**

**What is already known about this topic?**

The Surgeon General has concluded that there is a causal relationship between depictions of smoking in the movies and the initiation of smoking among young persons. The more frequently youths see smoking on screen, the more likely they are to start smoking; youths who are heavily exposed to onscreen smoking imagery are approximately two to three times more likely to begin smoking than are youths who are less exposed.

**What is added by this report?**

Previously reported declines in number of top-grossing movies with tobacco use has continued; however, the decline in the total number of tobacco incidents has not progressed since 2010. From 2010 to 2016, the total number of tobacco incidents in top-grossing movies increased, with a 43% increase occurring among movies rated PG-13.

**What are the implications for public health practice?**

Although there were fewer youth-rated films with tobacco incidents in 2016 than in 2010, total depictions of tobacco use has remained stable, concentrating such exposure in fewer films. Reducing tobacco incidents that appear in youth-related movies would prevent the initiation of tobacco use among young persons. An R rating for movies with tobacco use could potentially reduce the number of teen smokers by 18% and prevent their premature deaths from tobacco-related diseases.

## Footnotes

* Ratings assigned by the Motion Picture Association of America (a trade organization that represents the major movie studios) include the following: General Audiences (G): all ages admitted; Parental Guidance Suggested (PG): some material might not be suitable for children; Parents Strongly Cautioned (PG-13): some material might be inappropriate for children under 13; and Restricted (R): under 17 requires accompanying parent or adult guardian.

† https://smokefreemovies.ucsf.edu/.

§ Two common methods used to count smoking incidents in movies are to count the number of scenes in which tobacco use occurs or to count the number of cuts in which tobacco use occurs. Despite the difference in methods, both metrics have consistent results and are valid for comparing the results across ratings, years, companies, etc.

¶ https://smokefreemovies.ucsf.edu/sites/default/files/All%20tobacco%20depiction%20policies%200916.pdf.
