# *Erratum*: Vol. 66, No. 26

**DOI:** 10.15585/mmwr.mm6639a4

**Published:** 2017-10-06

In the report “Tobacco Use in Top-Grossing Movies — United States, 2010–2016,” on page 683, the last sentence of the third paragraph of the Discussion should have read “During 2010–2016, approximately 24 states awarded approximately **$1.7 billion** in public subsidies, such as tax credits, to productions of movies with tobacco incidents, including youth-rated movies.**”

